# Socio-environmental impacts of hydropower construction in Burundi

**DOI:** 10.1016/j.heliyon.2024.e40084

**Published:** 2024-11-02

**Authors:** Jean Marie Ndayiragije, Athanase Nkunzimana

**Affiliations:** a College of Hydraulic Science and Engineering, Yangzhou University, Yangzhou 225009, China; b Faculty of Engineering Sciences (Department of Civil Engineering and Environment), Burundi University, B.P 2720, Burundi; c Department of Geographical Sciences, Environment and Population, Bujumbura, Burundi University, Bujumbura P.O. Box 5142, Burundi

**Keywords:** Hydropower, Socio-environmental impacts, Sustainable development, Burundi

## Abstract

Hydropower serves as a very important element of the power system all over the world. And it has positive impacts on both economic development and on slowing down climate change related events such as floods and hydropower do not directly emit greenhouse gas which are ones of the main challenges facing humanity in the world. However, apart from its advantages, there are also various disadvantages of hydropower mainly related to its impacts on natural environment. This article overviews, presents and discusses various socio-environmental impacts of hydropower construction and operation in Burundi. Also, the study classifies direct and indirect impacts of hydropower as well as proposing their mitigation strategies. In this article a crucial aspect that is discussed is a new look at the installed capacity in terms of energy production and the presentation of insufficient generation capacity in Burundi which is one of the Sub-Saharan African countries still with a constant problem of lack of energy. The major negative impacts are changing in water quality, relocation of people, changes in the structure of the land and aquatic communities, loss in agriculture production, landslides and slopes destabilization and changing in climate. Another relevant aspect related with the design and construction of hydropower dams is that there are possibilities of creation of low flow reaches, downstream the reservoir, Therefore the river stretches should be remained with a minimum water flow to lessen the potentialities of the occurrence of hydrological drought and ecological damages.

## Highlights

Hydropower construction can.

• Negatively affect people through relocation and reduced agriculture production.
• Supply energy production and their reservoirs help in floods control in the downstream community.
• Exacerbate climate change related events, soil erosion and lead to the degradation of land.
• Create environmental issues like water resources contamination and ecosystem problems linked to the low flows.
• Have considerable positive and negative impacts in the local communities.

## 1 Introduction

Hydropower is the most commonly known source of renewable energy in the world, and hydropower generation does not directly emit air pollutants. Due to the role hydropower plays in energy sector and their economic impacts, there are more than 3700 hydropower stations with a capacity of at least 1 MW including planned or under construction in the world, mainly in the countries with emerging economies [1,2]. But, the construction of dams, reservoirs, and the operation of hydroelectric generators can significantly affect the natural environment and causes various impacts on the ecology [3]. The development of hydropower has been judged to cause many social and environmental impacts like triggering biodiversity loss, loss of habitat, degradation of land and forcing indigenous communities to relocate [[4], [5], [6], [7]]. From ecological point of view, the recent study indicated that hydropower development is seriously impacting areas of high freshwater megafauna in the regions such as South America, South and East Asia, and Balkan [8]. It has been estimated that more than half of the existing large rivers in the world has been influenced by damming [9], and the situation is worsening and the number will increase to 93 % by 2030 [10]. Though the report on hydropower development in Norway, indicated that hydropower has a large positive impact on national economy and can create tremendous opportunities in that sector [11], and the development of both large and small hydropower has received more attention in different regions of the world, especially in developing countries [[12], [13], [14], [15], [16]], however hydropower is not impacting all regions equally and its negative environmental impact often hit the headlines, especially large ones [17,18]. For instance, the assessment of potential socio-economic dynamic of Oyan dam between 1980 and 2016, in Nigeria, and the results criticized hydropower dam to have adverse effects on health of local population and downstream communities, and indicated that few people were actually benefiting or delivering their livelihoods from the dam [19], while a social survey of 160 households in four villages in central Laos was conducted to evaluate the impacts of hydropower projects and the findings shows that mostly were positive among the four downstream communities and hydropower was found to have the potential to be a major employer in downstream communities [20]. To have access to reliable and affordable clean energy is a key component for achieving sustainable development and clean cities. However, in Burundi hydropower which is the main source of clean energy is less represented, about 96.6 % of the total energy consumption is in the form of wood and charcoal burning while electricity is only 0.6 %, and 2,7 % and 0.1 % represent petroleum products and others, respectively [21]. Among the available sources of energy, hydropower is considered as the main source of clean energy, but it is of great importance to know its societal and environmental impacts as well as to promote the measures that reduce its construction and operation related environmental impacts [22]. Recently, Sun [23]taken the case of Three Gorges Dam in China to investigate the environmental influences of large hydroelectric dam construction and the results of the study indicated that the construction of dam has adverse effects on the regional climate and can lead to a considerable depletion of biodiversity. In a support with that, larger hydropower dams built along the Mekong River, 10.13039/100022984Amazon River, and the Congo River Basins, leading to both environmental problems such as alteration of people's livelihoods, displacement of thousands of people and affecting agriculture and food systems [24]. To the concerns about the possible effects of hydropower, Ni et al. [25] analyzed the economic impact of large hydropower projects in China, and the results suggested that hydropower development boost economic growth in most sectors such as health and education in the projects region, while some other sectors will suffer a loss in output because of the substantial real wages. And in 2010, the International Centre for Environmental Management (ICEM) [26], evaluated 12 proposed hydropower dams for the mainstream in the lower Mekong Basin. The study estimated that in case those projects are implemented would increase power security, navigation conditions for large vessels, foreign investment and economic development for certain sectors, but at the same time, they would generate adverse impacts on ecosystem functions, hydrological dynamic, fisheries, agricultural land and social system. And the examination on the effects of hydropower in 5 regions (Asia, Europe, Africa, North America, and South America) of the globe, indicated that the constructed hydropower dams associated with increased GDP in North America and in urban areas in Europe, but with decreased GDP, urban land, and population in the global south, and in reduced greenness in Africa in nearby areas [27]. In Sub-Saharan African countries including Burundi most people depend on natural ecosystems for wellbeing, livelihoods and tourism [28,29]. However, in these developing countries, natural ecosystems are threatened by natural phenomena especially climate change related events on one hand, and anthropogenic activities including mining, construction among others on the other hand [30]. As far as construction is concerned, dams and reservoirs have been constructed for many purposes such as flood control, irrigation, water supply, navigation, recreation, and aquaculture, among others. Hydropower dams are seen as an approach to alleviate the negative impacts of hydrological related disasters such as severe flood and drought that are likely to increase their intensities in the coming decades due to the rapid growth of the population and changing in climate [[31], [32], [33]]. Despite the role played by hydropower dams and their benefits, the construction of dams and reservoirs also have been criticized for their negative impacts on local population, natural ecosystems, and the surrounding environment. For instance, the study indicated that, the construction of hydropower dam reservoirs of Amerti and Neshe in the Northern Ethiopia increased food insecurity by 14.6 % [34], and the study on impact of 33 large-scale hydropower dams' construction in the global south, indicated that natural, human, social, and financial capital were negatively impacted, whereas the physical capital was often positively impacted [35]. Hydraulic infrastructures such as hydropower and its associated structures have profound socio-economic impacts on people from community all the way up to the national level. For example, dams can regulate and modify water flow in given river, which provide a benefit to the society like helping in flood control, but damming can threaten the riverine natural ecosystem by disrupting the movement and migration of aquatic organisms, increasing the risk of pollution as well as leading to the riverine habitat degradation [36]. And the study by Qicai Lin on how dams influence the river ecosystem, the construction of dam was regarded to have various disadvantages to the river basin such as substance sedimentation, reducing residential areas and causing diseases to human being. regarded as a barrier to fish spawning migration routes causing a decline in species diversity and abundance [37]. Hydropower provides electricity to many growing urban economies and emerging industrial, but also associated with social inequities between beneficiaries and disadvantaged of the dam project. For instance, the recent study by Schmutz Rita on how large hydropower impacts local social in the Amazon [38], the results indicated that, there is an increasing homicide among the young male people especially during and after construction, resulting in the significant loss of human capital. This means that, the impacts of hydropower dams are not limited to positive, they have also been found to cause environmental problems such as accumulation of sediment, disrupting hydrology of the rivers and their tributaries and social problems including delocalization of people and changing their livelihoods [39,40]. For instance, hydropower dams and reservoirs can significantly impact the local communities include energy generation, job availability, livelihood change, relocation of resident, among others [27,41]. The study on sustainable hydropower during 21st century conducted by Moran et al. [24], discussed and stated that the sector of hydropower should not only focus on the production of energy, but also incorporates the social and environmental negative effects caused by dams and reservoirs and taking climate change into consideration. The biophysical impacts including modification of both hydrological processes and the ecological system principally on biodiversity, and transformations in land cover and land use [42,43]. Many scientists from different disciplines, such as environmentalists, hydrologists and agriculturalists have paid more attention and conducted many studies on how hydropower projects pose tremendous impacts on the natural environment and leading to the degradation of land [20,[44], [45], [46]]. While the study used econometric methods to evaluate the relationship between country-level socioeconomic indicators and the development of hydropower for 56 hydropower plants built between the period of 1991 and 2010 in Brazil [47], and their study found that hydropower projects have positive effects on the economy but in short period of time (less than15 years) and highlighted that hydropower projects based on local long-term socio-economic development should be doubted. Concerning the energy sector in the east African countries, the World bank has recently reported that, it is only 10.2 % of Burundi's population that is having access to electricity https://www.worldbank.org pres-release›2024/01/10. “Every mouth get food to eat and every pocket has money it” said the president on his coming to the power, to improve the living condition of people and to reach the agenda of making Burundi a developed country in 2060, Burundi needs a better accessibility to renewable energy thus the construction of many of/or large hydropower stations to generate energy. Though, there is emerging hydropower construction in Burundi, and Burundi is one of the sub-Saharan countries highly vulnerable and exposed to climate change related events such as droughts [[48], [49], [50]], landslides [51], and floods [52], but the socio-economic and environmental impacts associated with the construction of hydropower dams and reservoirs are not well studied and documented. Therefore, the development of all hydroelectric projects in Burundi should present a comprehensive and well documented environmental impact assessment before the construction or operation approval by the National Environmental Agency, the potential environmental effects should be well studied as well as the proposition of their mitigation strategies and measures. Moreover, before the approval of the assessment of environmental impact, it is indispensable to implement the Public Audiences in order to hear and evaluate the local community's opinions regarding the construction of hydropower dams and reservoirs in the particular region. Thus, these situations motivated the authors to consider this investigation in order to provide necessary information on impact of hydropower construction in Burundi. Therefore, this paper tries to fill the gap by overviewing both negative and positive effects of hydropower construction in Burundi and presents the cause-consequences relationship between the development of hydropower projects and land change, as well as illustrating its direct and indirect impacts on land use and highlights their mitigation strategies. This study will serve as references for the future studies and as assistance to the decision makers to optimize the advantages of hydropower and minimize the negative socio-environmental impacts of large-scale hydropower construction. Furthermore, it will help water resources managements and environment protection decisions makers as well as the government in the execution of displacement rules and regulations for the affected communities during the implementation of hydraulic or other public infrastructures.

## 2 Location of hydropower data and methods

### 2.1 Location of hydroelectric generation in Burundi

In Burundi, both large and micro hydropower are the main sources of energy. As of 2010 decade, the interconnected network power production comes from two national hydroelectric power plants Mugere and Rwegura, and some smaller power plants in addition to the two regional power stations of Ruzizi 1 and 2. Burundi on its way of development together with its rapidly growing population, there is high demand of energy and water for industrialization and due to the fact, most of Burundians involved in small businesses that require electricity. In order to boost the national power generation, there are four hydropower projects three under construction and one recently starts its operation by which Burundi will more double the already installed capacity (which is 39 MW). The following table illustrates operational and under construction hydroelectric stations across Burundi, rivers on which they are installed and their corresponding basin.

**Fig. 1:**
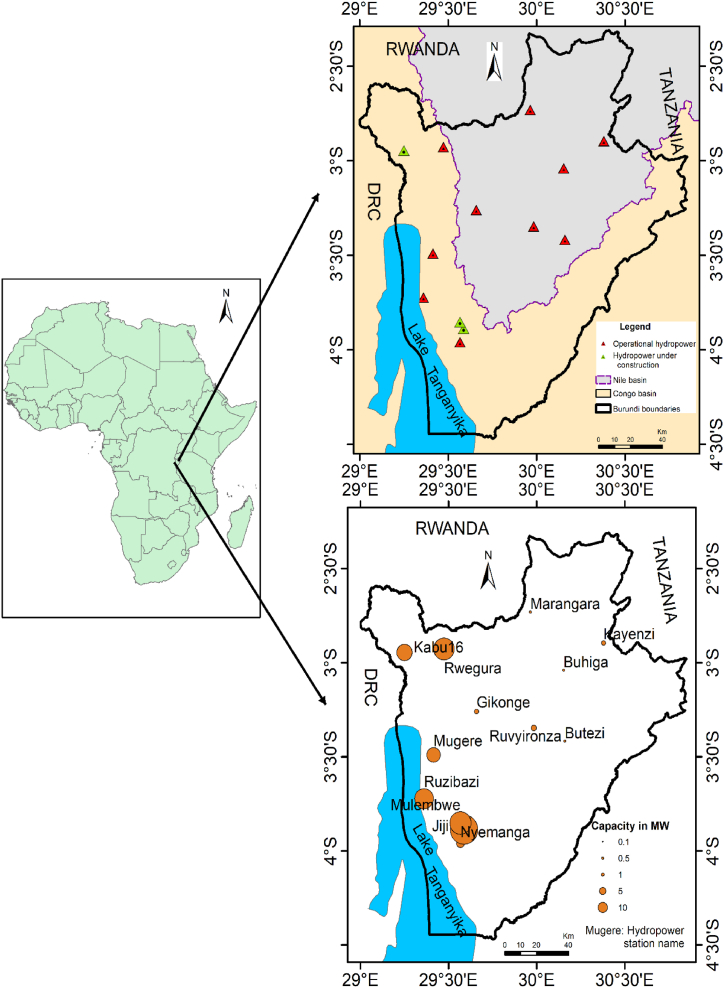
Map of Africa (Left) with geographical location of hydropower in Burundi by hydrological basin (Right up), and presentation of hydropower stations according to their power generation capacity in terms of Megawatt (MW), (Right down).

The majority of the hydropower stations in Burundi are installed in the sub-catchment of the Ruvubu river (Nile basin) while the largest are installed in the sub-catchment of Lake Tanganyika (Congo basin) as shown in Fig. 1. The two sub-catchments are already exposed to the environmental menaces due to the majority of the population of this country settling in rural areas depending on natural resources available from the catchment in addition to the rainfed agriculture for survival.

### 2.2 Methodology

To overview socio-environmental impacts of hydropower construction in Burundi, the authors search for articles exactly related to the subjects of hydropower construction, and keywords such as “hydropower, dams and reservoirs, socio-economic impacts, biodiversity or ecosystem” were used to search for titles, abstracts and keywords. The searching engine such as PubMed, Google scholar, and Web of Science were used to search for documents published as journal and conference articles, book chapter and reports. In addition to those computational searches, manually search, experience and field investigation were conducted. And during the field investigation a semi-structured interview with the authorities from the communities where hydropower construction projects have been implemented and are going on was conducted in order to explorer what the local population think about hydropower construction. During the discussion between authors and the authorities of the communities was in local language (Kirundi) to avoid English barriers (see Table 1).

## 3 Results and discussion

There are various socio economic and environmental impacts linked to the construction of hydropower or any other hydraulic infrastructure. The most relevant negative and positive socio-environmental impacts related to the construction of hydropower plants are classified in Table 2 and described as follows.

**Table 1:** List of installed and under construction hydropower stations in Burundi, including their capacity according to the Water and Electricity Generation and Distribution Corporation (REGIDESO).

Hydropower stations	River	Drainage basin	Power generation capacity (MW)	Types
Already constructed hydroelectric				
Rwegura	Gitenge	Tanganyika	18	Storage
Mugere	Mugere	Tanganyika	8	Run of the river
Nyemanga	Siguvyaye	Tanganyika	2.8	Run of the river
Ruvyironza	Ruvyironza	Ruvubu	1.275	Run of the river
Gikonge	Mubarazi	Ruvubu	0.85	Run of the river
Kayenzi	Kayenzi	Ruvubu	0.85	Run of the river
Butezi	Sanzu	Ruvubu	0.24	Run of the river
Buhiga	Ndurumu	Ruvubu	0.24	Run of the river
Marangara	Ndurumu	Kanyaru	0.24	Run of the river
Ruzibazi	Ruzibazi	Tanganyika	15	Run of the river
Under construction hydroelectric				
Kabu16	Kaburantwa	Tanganyika	20	Run of the river
Jiji	Jiji	Tanganyika	31.5	Storage
Mulembwe	Mulembwe	Tanganyika	19	Storage

**Table 2:** Classification of negative and positive impacts associated with hydropower construction in Burundi.

Negative Impacts	Positive Impacts
Relocation of people. Changes in climate. Wide spread of certain diseases. Contamination of water resources. Thermal pollution. Fragmentation of rivers and ecosystems. Land degradation. Reduction in agricultural production. Induced seismicity. Changes in the hydrological regimes. Effects of flow regulation on general ecosystem.	Job availability. Electricity production. Improvement of water quality. Other advantages uses of reservoirs and dams.

### 3.1 Negative impacts that result from hydropower generation

#### 3.1.1 Relocation of people

Depending on the reservoir's designed capacity, the construction of hydroelectric plant requires a significant area of land. There is need of roads in order to reach the site, parking of cars and machines, housing and offices during the construction and the operation phases. All of these requirements might lead to the significant number of people to be relocated from their own properties (land) in addition to where reservoirs and other related infrastructures are planned. This might constitute an undeniable disadvantage in case the region is very densely or if is constituted of arable land for agriculture. Relocation of people does not only have direct effects on the natural environment and agriculture, but also on the education of children and mental health for some people, especially when relatives or friends are separated. Moreover, the relocation of people leads to the wide expansion of cities and results in the changes of landscape, which often intensify the impacts of climate change on streamflow, topography, agriculture, and natural environment in general. Based on the summary report of environmental and social impact assessment for Jiji and Mulmbwe hydropower project, the total of 341 households with about 2237 people affected by Jiji project, and one primary school together with church which was situated near Jiji dam necessitated temporary relocation [53]. The following Table 3 shows how the construction of hydropower can result in permanent relocation of people which in return can affect social and environmental aspects of the surrounding community.

**Table 3:** Permanent relocation of people as impact of hydropower construction (source: Authors from on-site investigation).

Hydropower name	Site	Number of relocated houses	Public facilities
Jiji	Dam	18	1 tap water
Jiji	Penstock	13	1 Church
Jiji	Powerhouse	10	–
Mulembwe	Powerhouse	11	–
Mulembwe	Road	10	–

#### 3.1.2 Changes in climate

Hydropower is source of renewable energy which is not highly cost compering to other sources of energy, and is with a significant contribution to the reduction of greenhouse gas (GHG) emissions, and hydropower plants don't generate carbon dioxide since they are not burning fossil fuels. However, in the natural process climate change and development of hydropower interact each other [54]. Regarding the impacts of climate change on natural water resources, hydropower reservoir with large storage capacity can serve as an adaptative measure and more resilient to climate change [55]. But, the construction and implantation of hydropower dams and reservoirs might be associated with certain activities such as blasting of rocks which might result in depletion and pollution of groundwater. And groundwater pollution is increased by anthropogenic activities especially in developing countries [56], and every construction project definitely create both air and water pollution which will disturb local community's living conditions and climate in general [56,57]. The construction of hydropower and its associated infrastructures can cause the wide cutting of trees and result in deforestation which may leading to the wide death of certain organisms and/or affecting their reproduction, and the contamination of water resources. And the case of Burundi, during the construction of hydropower such as Ruzibazi, Kabu 16, Jiji and Mulembwe involved the use of explosives to blast the rocks for tunnels drilling. This may affect both atmosphere and the surrounding environment by producing heat, hazardous air and the waste deposit from blasted rocks. And all of these activities can easily exacerbate the occurrence of climate change related incidents such as soil erosion, land degradation, and droughts especially groundwater, hydrological and ecological droughts.

#### 3.1.3 Wide spread of certain diseases

Water stored in reservoirs can become breeding grounds and a favorable environment for disease vectors such as snails and mosquitos which are vectors for Schistosomiasis and malaria, respectively [58]. This is true especially in the regions that are characterizing by tropical and inter tropical climate including Burundi where mosquitoes can take advantage of the slow flowing water to easily breed. Also, during the construction phase of both large or small hydropower reservoirs, too much dust pollution can be produced which can slowdown the growth and reproduction of plants on one side, and cause certain human's diseases such as flu, lung cancer, chronic obstructive pulmonary disease (COPD), and lower respiratory infections among others, which might increase the risks of dying to the workers on the other side. In addition, many workers in the project might be coming from several regions of the country or abroad and may bring vectors of contagious diseases to the local communities, and some factors that are disruption to the society such as, selling and consuming drugs, criminality, prostitution, teenager pregnancy and wide transmission of HIV. Large-scale hydropower projects such as Huangshi and Danling dams in China, Aswan dam in Egypt, Gezera-Managil dam in Sudan, and Melkasadi dam in Ethiopia, are typical example of dams' projects that their construction have resulted in the rises or recurrence of Schistosomiasis [59].

#### 3.1.4 Contamination of water resources

There are no natural resources which are precious than water resources due to its significant role in the growth of economy and sustainable development of the World. However, there are several human activities that leading to the pollution of water resources. Hydropower dams and reservoirs for instance, can lead the submersion of large areas of land which may contribute to the accumulation and release of nutrients, for instance phosphorus and nitrogen originated from the flooded biomass. Hydropower dams degrade the quality of water along the rivers [60,61]. For instance, in water that flows downstream from the dams the oxygen is reduced, which significantly harms many aquatic organisms on one side. On the other side, the reservoirs above dams are highly prone to the harmful plants such algae. And some kinds of algae plants are poisonous and they can have negative impacts on the health of people in case consumed. Moreover, when algae burst out, water quality suffers from poor oxygen conditions, and the characteristics of clean water will be affected by the existence of algae. Due to the fact that, the presence of algae can both significantly and negatively impact the color, taste and odour of water, life of fish and other organisms of the water will be almost practically impossible and water will no longer be appropriate for human consumption and other uses. The long existence of reservoirs allows the algal blooms, which can leach toxic metals such as the mercury from submerged soil to increase in the water system. This mercury will then start to biologically magnify within the food chain, eventually causing a health risk to the surrounding population that rely on water food source, especially fish [62]. During the construction phase of hydropower, there is considerable discharge of wastewater because of construction activities and domestic wastewater from the site's camps of workers [63]. Due to the difficulties in the management of the produced wastewater, water bodies near the hydropower construction site and the surrounding environment are sensitive to that wastewater which even affect the entire river basin. Since hydraulics infrastructures are needed for the development and operation of water resources, power generation and irrigation in order to meet the demands for energy and food, their implementation should refer to the governance, infrastructure, and water resources management measures necessitated to monitor and evaluate fresh water availability to meet the needs of both natural ecosystem and Human.

#### 3.1.5 Thermal pollution

Thermal pollution is defined as any deviation from natural temperature in a habitat and can range from elevated temperatures linked to industrial cooling activities to discharges of cold water into streams below large impoundments. The thermal pollution is problematic as much of stratification of oxygen. In a hydropower reservoir, thermal energy is highly accumulated in the top layers that are directly exposed or closer to the Sun, while the rest of the reservoir increasingly becomes colder. In other to generate energy in hydroelectric plant, water can then be drawn into the turbines through a penstock situated at the bottom of the reservoir leading to the changing in temperature of the water downstream. The construction of hydropower in Burundi is the main project that involve in the use of many diesel or petrol engines vehicles as well as construction machineries. The wastewater from washing of those machines and trucks might be warmer than the streams that they are flowing in, and then create thermal pollution [64,65], and regulation of hydropower itself can affect the thermal regimes mainly in the high head systems and reservoir capacity [66]. The extreme changes in temperature can directly trigger fatalities in aquatic populations [67], but even moderate changes in water temperature is not something negligeable because it can modify the metabolism, and both reproduction and growth of water organisms. Moreover, thermal pollution can also extensively impact the available freshwater resources and eventually influence the ecological systems.

#### 3.1.6 Fragmentation of rivers and ecosystems

The construction of hydropower can cause fragmentation of rivers, for example, the recent study on the impacts of hydropower dams on fragmentation of river indicated that the construction of many small hydropower can cause significant increasing of rivers fragmentation that will not be accompanied by a corresponding increase in hydropower in Balkan region [68]. But, in Africa depending on financial capability and because of small hydropower play in the rural electrification and providing assistance in reducing poverty [69], many African countries including Burundi implemented and continue to implement small hydropower to tackle energy problems [70]. Hydroelectric dams may block fishes from migrating to spawning areas, leading to a significant decrease reproduction numbers and reduced species population. Putting in palace fish ladders or installation of other corresponding structures such as barge or use of fish lifts could potentially lessen this issue. Reservoir flooding can cause permanent inundation, which also leading to the changes of forests, wetlands and other habitats of the surrounding river environment [71,72]. In addition to that, there is an occurrence of ecosystem disturbance in the riverbank's areas, which are commonly rich in ecosystem or biodiversity, due to support of natural flooding of a dam-free river. It is undeniable that dammed rivers play a key role in reducing both flood rates and consequences, but this has negative effects on the floodplains downstream especially the ones that are heavily rely on seasonal waters for agriculture and survival purposes.

#### 3.1.7 Land degradation

**Fig. 2:**
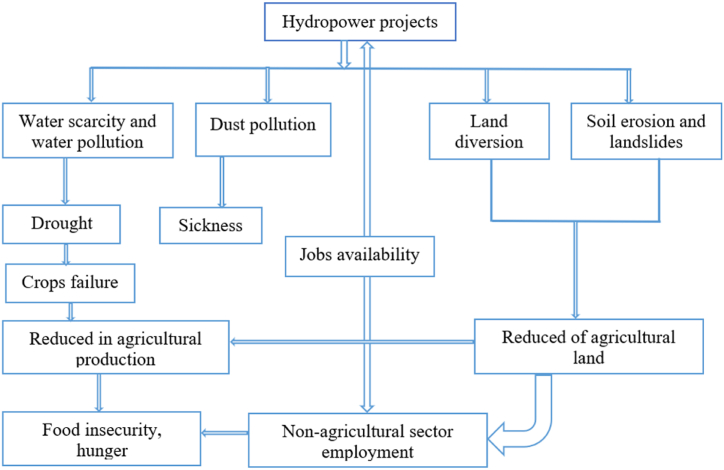
The cause-consequences relationship between the development of hydropower projects and land change.

Degradation of land is process in which the values of the biophysical environment is highly affected by a combination of human induced acting upon the land [73]. It is a challenge to have a common definition of land degradation, based on the fact that, what one group of people can see as a degradation of land while another group might see it as a greatest opportunity depending on the living conditions. For instance, farming in the region characterized by climatological and geographical conditions such as heavy precipitation and steep slopes respectively, would draw more attention to the researchers and scientists concerning the risks of soil erosion caused by rainwater. However, farmers could see the place as a suitable place for planting crops. No matter how people cannot see the degradation of land in the same way, hydropower projects significantly degrade land through the construction of dams, roads to access the sites, and power lines that causing soil erosion and landslides [74]. Burundi is small country with its tiny arable land for farming, and about 90 % of its population are relying on agriculture, traditional agriculture which relies on many factors such as forest and vegetation cover to feed their livestock for the production of manure which is used to fertilize the farming land. Degradation of land significantly affects agricultural production and leading to food insecurity. The following Fig. 2 illustrates the cause-consequences relationship between development of hydropower projects and change of land, and its effects on socio-economic aspects, while Table 4 shows the direct and indirect impacts linked to hydropower projects on land use and their mitigation measures.

**Table 4:** Direct and indirect impacts of land use for development of hydropower projects and mitigation measures. (Source: From on-site investigation conducted by authors).

Originality of land use	Current land use	Direct impacts	Indirect impacts	Mitigation measures	Power plant involved
Farmland, forest and vegetation.	Roads to access the sites	Loss of farmland	• Reduction in agricultural production • Food insecurity • Lower income to farmers	None	Jiji, Mulembwe
		Dumping of muck and covering of land	• Reduction in agricultural production	➢ Soil conservation ➢ Afforestation. ➢ Reclamation of land for agriculture.	Jiji, Mulmbwe
		Dust pollution	• Loss of crop quality and quantity • Wide spread of sickness	➢ Sprinkling of water especially in dry season. ➢ Ensure health care availability.	Jiji, Mulembwe
		Soil erosion and landslides	• Reduction in agricultural production • Disruption of marketing • Occurrence of natural disaster (drought in particular)	➢ Engineering works ➢ Planting trees and vegetation.	Rwegura, Jiji, Gikonge
	Excavation of Tunnel	Groundwater flows in the tunnel	• Decrease in the surface water level and depletion of wells. • Groundwater drought	None	Ruzibazi, Kabu 16, Jiji, and Mulmbwe,
		Water supply disruption	• Reduction in agricultural production. • Sickness linked to the lack of sanitation.	➢ Development of other sources of water for irrigation, and clean water for drinking.	Jiji
	Hydraulic infrastructures and other buildings	Loss of farmland	• Reduction in agricultural production	None	–

#### 3.1.8 Reduction in agricultural production

**Fig. 3:**
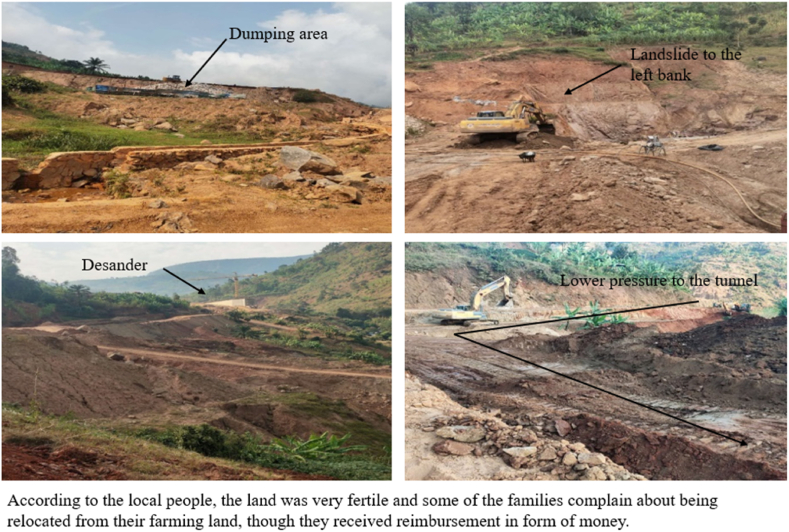
Pictures illustrate the farming land that was taken due to the construction of Jiji hydropower dam.

The construction of dams, reservoirs and hydroelectric plants require a big area of land and people might be relocated from their farming land which obviously result in lower production of agricultural product [34,75,76]. For instance, the ongoing construction of JIJI and MULEMBWE hydroelectric projects in Burundi, leads to a wide cutting of trees and different varieties of plantations were uprooted as shown in Table 5. Some families lose areas of plantation which are their main sources of income revenue and significantly contribute to the GDP of Burundi. The farmers received reimbursement in the form of money but the reimbursement money might not be enough to buy another arable land. For example, the dam and reservoir of Jiji hydropower and other related infrastructures and activities are situated and affecting the place called Gatakwe, “Gatakwe: meaning a land that is producing a lot of agricultural products” according to the local people, as shown in the pictures below, according to the local communities the land was more fertile to grow and produce many varieties of crops such as beans, casava, maize, bananas, onions, and groundnut. The construction of JIJI and MULEMBWE hydroelectric plants and their associated activities including roads, offices and stores as example shown in Fig. 3, about 5600 ha of land was affected. Therefore, there is a significant number of farmers saying that, they are living in the extreme poverty and complaining about their livestock not having pasturage. Moreover, the employment of a lot people who are in active ages, left the agricultural sector for old people, who are not able to cultivate the plants that will meet the communities ‘food demand.

**Table 5:** List of various plants in terms of quantity and areas of plantation affected due to the implementation activities of Jiji hydropower station. (Source: from the on-site investigation done by the authors).

Owner	Location	Plants name	Quantity	Area in m^2^
Hakizimana Cyprien	Nyabitanga	Grevilia Cassava	1 –	10
Bakinamugwanko Gordien	Nyabitanga	Bananas Cassava	5 –	13
Nshimirimana Claudine	Nyabitanga	Suit potatoes Maize Cassava	– – –	15
Hatunga Cyprien	Nyabitanga	Bananas Eucalyptus Suit Potatoes Cassava	11 1 – –	15 40
Ndikunkiko Elias	Nyakigo	Palm oil Guava fruit Bananas Eucalyptus Pineapple Cassava	3 6 1 2 2 –	613,37
Ndayisaba Boas	Nyakigo	Coffee trees Guava fruit Mango trees Bananas Grevilia Tangerine Palm oil Avocado Cassava	30 10 2 16 2 1 7 2 –	151,5
Total				857,87

#### 3.1.9 Induced seismicity

The construction of a dam across the river leads to impoundment of water which results in the reservoir induced seismicity depending on the geo-hydrological conditions of the area [77]. There are two ways or procedures that triggers the earthquake in structural of reservoir: One is that water stored in the reservoir generates water pressure through infiltration process and diffuse pores and reduces the normal stress, which result in the reduction of faults ‘friction and provokes the earthquake; second is both chemical and physical effects of reservoir water on the rocks, such as lubrication, stress corrosion, expansion, and final the rock becomes soft and leading to the destabilization of slopes and trembling of the rock masses. During the early times after water filling the above-mentioned effects can be noticeable, and might pose a serious menace to the regions that are sensitive or prone to earthquakes across the globe. These catastrophes could negatively impact dams and reservoirs, and lead to their failure which could result in the flooding of large areas of land, damaging downstream settlements and infrastructure, causing the loss of life, local biodiversity, and as well as reducing the biological genetic patrimony such as fauna and flora.

#### 3.1.10 Changes in the hydrological regimes

The construction of hydropower dams has major impacts on hydrological and alluvial regimes of rivers, mainly through the changes in frequency, timing, and magnitude of low and high flows, ultimately producing a hydrologic regime to significantly differ from the pre-impoundment natural flow regime. Natural water resources have been suffering from multiple transformation due to the increasing water demands in many regions of the globe. And among the changes caused by hydropower dams, the flow timing which can result in delaying the peak of the flood and affecting seasonal flows downstream [78]. It is undeniable that both dams and reservoirs alter the thermal and flow regimes of river [[79], [80], [81], [82]], for instance, the recent study demonstrated that both small and large hydropower facilities cause hydrological alterations which are attributable to hydropeaking [83]. Also, Penas and Barquin [84]established general patterns of hydrological alterations caused by dams on national level in Spain, and their findings indicated that the magnitude and direction of hydrological alteration depend on the natural flow class of the altered rivers. Since hydropower dams have been criticized to significantly elevate the rate of flow changes and water temperature [85], and freshwater provides fundamental services to human societies, these changes can negatively affect the living conditions of the people that rely on water flowing in the rivers for their various activities. Given the case of Burundi, where there are many people use flowing water of the rivers for irrigation of their crops, feeding their livestock, washing clothes and housing cleaning, therefore the downstream user of water can be affected by hydropower dam. Moreover, the reduced water flow in the river-floodplain connectivity and loss of linked ecosystem services of the altered river can cause major ecological implications downstream parts.

#### 3.1.11 Effects of flow regulation on general ecosystem

Hydropower dam requires the regulation of flow, and the regulation of flow has effects on hydrological regime of the river such as influencing water chemistry and the dynamics of sediment which in return affect the natural ecosystem of the river. For instance, the recent study that investigated the conditions of minimum water flow at the Ruzizi Hydropower dam in terms of land use management and ecological impacts, indicated that rivers system require water to maintain its hydraulic and ecological functions, otherwise the significant disturbance of flowing regime can prevent the rivers from providing their ecosystem services and cause the disruption of riparian communities [86]. In given river, the low flows can cause the native vegetation and trees to dry, and result in considerable negative impact on ecosystem. Those trees can maintain the stability of river's banks, providing the right food for various insects, and their falling leaves can provide food for organisms of the river. Moreover, during the dry season, in some of the rivers the low flows is nearly eradicated by damming, as results aquatic plants may not be able to push flowers for pollination. Since poor flow control has been acknowledged to be the most devastate species that produce semi-plaggic eggs [87], much more attention should be paid in regulating the flow during the operational phase of a given hydropower.

### 3.2 Positive impacts result from hydropower generation

#### 3.2.1 Job availability

In developing countries, where the majority of the population is not educated and the level of joblessness is high, usually face underemployment related problems, which might result in the increasing of criminal activities. The installation of a new large hydropower plant creates a clear expansion of working posts offer during the construction phase and result in reduction of unemployment. This might have a significant socio-economic impact starting from the local families up the national level. More educated people especially the youth withdrew from other sectors such as agriculture, commercial among others and took up employment with the construction companies, because they get a better livelihood option compering to the other sectors.

#### 3.2.2 Electricity production

According to the world bank, developing countries are suffering from a sustained lack of electricity. Taking the Sub-Saharan Africa (SSA) countries, including Burundi which are highly vulnerable to energy crisis, whereby is only an estimate of 31 percent of the population has access to electricity, and approximately 600 million of people are not having access to sustainable electrical energy and clean water [88]. In SSA countries the price of electricity is very expensive, and the average power cost at $0.12/kWh, which is almost twice higher compared to other developing countries. The generation of hydroelectric power that can replace diesel based thermoelectric and other sources of energy such as charcoal and wooden fire, is considered to be the most advantageous at both environmentally and economically point. And in terms of business, hydropower plants may last for several decades, and the more they last for a long the more they have long economic impacts. Compering to the cost of fossil fuels, such as natural gases, coal, and oil, the operation of hydropower plant will definitely be with lower cost.

#### 3.2.3 Improvement of water quality

Since hydropower dams lead to the lessening of turbidity, nutrients sedimentation and eutrophication process downstream, this can significantly improve the quality of water resources. For example, the study on the impact of small hydropower plant on water quality in Poland [89], revealed a significant improvement in quality of water below hydropower plant. Since in Burundi there still a considerable number of people which is not having access to clean water, using rivers’ flowing water for different purposes such as irrigation, washing clothes and feeding their livestock. The improvement of water quality in the below part of the hydropower dam can serve as an important advantage of hydropower for many rural families in Burundi.

#### 3.2.4 Other advantages uses of reservoirs and dams

The natural water resources such as rivers and lakes, water varies overtimes depending on season. During very rainy season or when mountain snow is melting, water level rises in rivers and sometimes overflow their banks. Reservoirs and dams can therefore, limit the amount of water to continue downstream and result in flood controlling. Though, in hydropower projects reservoirs are created to store water for power generation, they often serve as recreation facilities and may become tourism places. Reservoirs can also serve as water allocation for different purposes such as industrial and domestic use and can be used for farming as irrigation purposes, which have a valuable impact in many regions across the globe in food security.

## 4 Mitigation of socio-environmental negative impacts related to hydropower construction

It is undeniable that hydropower has both positive and negative effects on societies. Therefore, there is need for measures and strategies that could alleviate socio-environmental negative impacts associated with the implementation of hydropower, and this including human rights to safe drinking water and indigenous rights in the context of hydropower development [90]. It has been highlighted that beside the reduced finances and lack of investment, there other many factors such as environmental and social costs, lack of transparency, low stakeholder participation have led to a decline in implementation of large dams [91]. And it has been found that dams utilized to generate hydropower also fragment rivers and cause considerable sociological and ecological impacts [92]. Hydropower plant demands for huge deforestation, which would directly result in the destruction of critical ecosystems, large migration of people at downstream, and cause other disasters such as landslides and flash floods [93]. Then, the following measures are underlined and encouraged: 1. Sustainable water resources management projects are needed in place, and strict policies to lessen socio-economic and environmental impacts during and after construction of hydropower; 2. Collaboration between decisionmakers on the policies that promote renewable hydropower energy and those that their priority is the conservation of ecosystems wetlands and rivers in particularly; 3.The alteration in the flow characteristics of river and its water quality can threaten aquatic life, especially the diversity of fish, it is crucial to locating the projects in areas with lower biodiversity and natural ecosystem services values, and considering the rivers with lower ecosystems services downstream and that already have regulated water flow [94]; 4. The construction management should be strengthened and engage all workers to be more responsible and participating in environmental protection, and strict measures such as drainage systems and waste disposal should be sufficiently provided in order to avoid the wastewater from the area such as batching plants, and organic waste from dining hall and kitchen to straitly flown into rivers or lakes; 5. Strict control of machines, trucks and vehicles to prevent the leakages of mazut, gasoline and other oils for the engines, avoiding the spills of hazardous materials, chemicals and hydrocarbons, as well as regularly training of machines' operators to avoid accidents; 6.Back fill all necessaire places on site that were excavated or damaged by the machines and trucks to avoid rainwater stagnant, rehabilitate by practice the method of greening such as planting trees and vegetations; 7. Reimbursement to the relocated people from their land, should not be given in terms of money only, but considering of giving other land especially the rural farmers that solely depend on agropastoral activities; 8. Natural environment protection is a major concern for everyone, and the recent by Castro-Diaz et al. indicated that, the lack of local communities participation in decision-making can have negative impacts on the people's natural, social, human and financial capital aspects, therefore the integration of local indigenous people while implementing the construction of hydropower dams and reservoirs is highly recommended in order to lessen negative impacts linked to the construction activities.

## 5 Research gap and recommendation

The available literature can constitute valuable insights in many aspects concerning the construction of hydropower dams and reservoirs and their impacts on both society and environment. However, there is no significant documentation regarding the opportunities and challenges connected to the development of hydropower in Burundi. In Burundi, financial and political motives are the major dominant for the planification of hydropower and its connected structures, but the country is susceptible to climate change and weather variability related events. For instance, in 2017 Regideso reported that the Rwegura hydropower reservoir's water level declined from 12m to 9,56m because of drought as results many cities in Burundi experienced a severe shortage of electricity [95], and it is projected that streamflow will decline in the Rwegura catchment and this decreasing in the streamflow will definitely have negative impacts on hydropower production [96]. Therefore, in the selection of new hydropower sites, the planning and management of land use, climate change related events should consider long-term scientific data to tackle how hydropower construction and operation can exacerbate climate change and how climate change affects hydropower production. In Burundi limited research has been undertaken to comprehensively investigate societal and environmental impacts of hydropower, thus the limits available documents on this topic. And given the fact that, there is emerging development of hydropower projects in the Tanganyika sub-catchment and this can have impacts on the hydrological regime in the basin [97], many studies that can use the indicators of hydrological modification method to assess the impacts of dams on hydrologic regime are highly recommended. There is a need for more studies on socio-environmental impacts of hydropower and it is imperative to initiate researches that set up models to optimize reservoir operation rules to sustain during drought incident, more over it is recommended to conduct comparative studies to see whether hydropower is more negatively/or positively affecting the local population, especially in the rural communities.

## 6 conclusion

Hydropower development and other hydraulic infrastructures are putting pressure on water resources particularly rivers and other natural ecosystems that provide an important habitat to the freshwater fish at all levels. While dams can provide multi services including water supply and security, prevention of flood, and energy supply, they can also considerably threaten freshwater organisms and limit their capabilities to reproduce. Since there are millions of people that are highly dependent on agricultural production to survive and the electricity is needed for almost in all human activities worldwide, especially in developing countries including Burundi where many people involve in small businesses that require the use of electricity. Therefore, more hydraulic infrastructures such dams and reservoirs are likely to be more built in sub-Saharan countries for different purposes such as the practice of irrigation and energy production to meet the demands of people and making a transition from the high shortage of food and the deficit of energy to the food security and sufficient energy respectively. To understand both negative and positive socio-environmental impacts of hydropower development within a given river basin, is crucial step in the implementation of measures and identifying solutions that needed to produce electrical energy while minimizing the negative impacts on essential natural resources such as streams and forests, as well as on the wellbeing of the local population and avoid the exacerbation of climate change. This study overviews the negative and positive socio-environmental impacts of hydropower projects in Burundi, direct and indirect impacts were classified and their mitigation strategies were proposed. By concluding the study suggests that the government, civil societies, water resources management policy makers, water users and scientific community are called to deeply involve in the relevant discussions concerning the implementation of hydropower projects, proactive measures and activities that protect the natural environment and resources such as management and operation of water resources, back fill all halls that are excavated during construction, and compacting the soil material of the dumping areas to avoid erosion. Moreover, this overview recommends more studies with the employment of models that can assess how natural environment is negatively affected by the construction of hydropower and/or other hydraulic infrastructures.

## CRediT authorship contribution statement

**Jean Marie Ndayiragije:** Writing – review & editing, Writing – original draft, Visualization, Validation, Software, Resources, Methodology, Investigation, Formal analysis, Data curation, Conceptualization. **Athanase Nkunzimana:** Visualization, Formal analysis, Conceptualization.

## Data availability

All data and information used for this study are within the manuscript.

## Funding

Not applicable.

## Declaration of competing interest

The authors declare that, there is no conflict of interest.
